# Low increase in phenylalanine tolerance during pregnancies in PKU woman with high prepregnancy BMI and postconceptional initiation of diet: A case report

**DOI:** 10.18502/ijrm.v17i10.5302

**Published:** 2019-11-07

**Authors:** Joanna Żółkowska, Kamil Hozyasz, Ph.D.

**Affiliations:** ^1^PKU Polyclinic, Institute of Mother and Child, Warsaw, Poland.; ^2^Institute of Health Sciences, State School of Higher Education, Biała Podlaska, Poland.

**Keywords:** Phenylketonuria, Pregnancy care, Phenylalanine.

## Abstract

**Background:**

Women with untreated phenylketonuria (PKU) are at an increased risk to have offspring with multiple abnormalities due to teratogenic effects of hyperphenylalaninaemia. Treatment goals include blood phenylalanine concentrations between 120 and 360 µmol/L, however, there are limited pieces of evidence for the practical management of pregnant PKU patient and prediction of phenylalanine tolerance changes during a course of pregnancy.

**Case:**

We report the case of a mother with classical PKU (p.R408W/p.R408W) and the course of her two pregnancies with low phenylalanine tolerance increase (347mg and 227mg) despite the rewarding collaboration with a nutritionist.

**Conclusion:**

This case report does not confirm the observation that a very low phenylalanine tolerance increase in pregnancy of PKU patient is a marker of coexisting PKU-affection in fetus.

## 1. Introduction

Classical phenylketonuria (PKU, OMIM 261600), caused by a deficiency of the biopterin-dependent cytosolic enzyme phenylalanine hydroxylase (PAH, EC 1.14.16.1), is the relatively frequent inborn error of metabolism. Over the last half century, population newborn screening has made PKU one of the most spectacular examples of diseases whose manifestations can be prophylactically managed via early intervention (1-3). In PKU, the essential aromatic amino acid phenylalanine (with the formula C${}_{9}$H${}_{11}$NO${}_{2}$, Phe), cannot be converted to 4-hydroxyphenylalanine (tyrosine) and therefore pathologically accumulates. Dietary intervention restricting Phe intake has converted PKU to a disease with essentially normal neurodevelopment and social integration (1-3). Phe tolerance (mg/day) is the amount of this amino acid in food that those with PKU can eat and maintain acceptable blood Phe levels. Clinical determination of Phe tolerance relies on the systematic assessment of blood Phe levels in relations to Phe intake from detailed menus provided by the patient. A protein substitute free of Phe, but nutritionally complete in all other amino acids, minerals, vitamins, and trace elements is an essential part of dietary management.

Pregnant women with PKU need appropriate management to avoid raised levels of Phe with teratogenic properties, leading to a host of birth defects, especially microcephaly and congenital heart disease (so-called maternal PKU syndrome) (2, 3). Therefore, having a pregnant patient with PKU is an important public health problem with direct impacts on the offspring's health and quality of life (4). With that in mind, PKU women intending to get pregnant are advised to use a restricted low-Phe diet that starts at least 4 wk before conception to prevent teratogenic effects of increased Phe. However, the Phe intake should ensure the necessary amount of the amino acid needed to support the protein turnover for current body mass of mother and fetus/es. During pregnancy, the Phe tolerance may improve to some extent because of the increased fetal activity of PAH. It should be noted that there are limited pieces of evidence for the practical management of pregnant PKU patient (3, 5, 6). For example, the prediction of phenylalanine tolerance changes during the course of pregnancy is not clear.

The purpose of this case report is to describe phenylketonuric woman with a very low increase of Phe tolerance during pregnancies.

## 2. Case Presentation

A Caucasian patient of Polish origin was diagnosed with classical PKU at one month of age and remained on phenylalanine-restricted diet until the age of 17 years (IQ 105), however, her metabolic control during adolescence was not fully satisfactory. She became a food technologist. The patient has had two pregnancies and did not follow phenylalanine-restricted diet prior to the conception in both and the cases; her whole blood phenylalanine concentrations on normal diet were above 1,200 µmol/L. She returned to dietary phenylalanine restriction when she was almost five wk pregnant and worked with a metabolic dietitian (J.Ż.) to stabilize blood Phe levels. Dietary treatment included supplements and nutritional mixtures specifically devised for pregnant PKU patients (Table I), which were taken in 3-5 doses per day. Whole blood Phe concentration has been measured using the tandem mass spectrometer technique (Ms/Ms) at least twice a week and a target range of phenylalanine of 120-360 µmol/L was applied. If the blood Phe concentration remained above acceptable range, Phe intake was slowly decreased. The Phe intake was increased without delay if the blood Phe concentration was ≤ 120 µml/L (3). The Phe tolerance was empirically determined based on the analysis of fluctuations of the patient's blood Phe concentration in relation to dietary Phe intake. Apart from the metabolic physicians and dietary care, the pregnancies were managed in the normal way by the obstetricians. It should be noted that the patient's prepregnancy BMI was high (Table I). Moreover, her mean total weight gain for pregnancies was 14.5 kg.

There were no obstetric complications throughout the pregnancies and the babies were born by normal delivery, see Table II for birth measurements. None of the infants had PKU or any degree of persistent hyperphenylalaninemia. The neurodevelopment of offspring was normal over a follow-up periods of at least three years.

**Figure 1:**
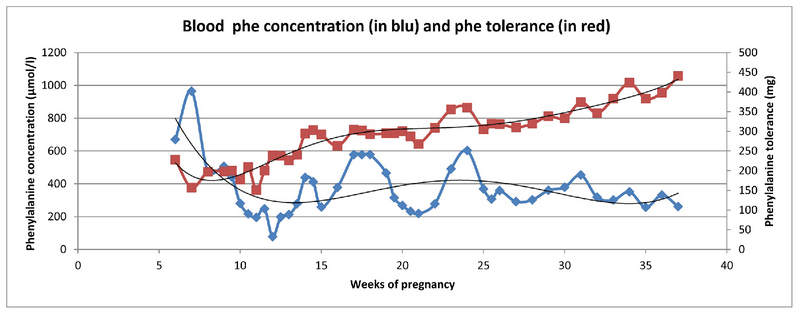
Phenylalanine tolerance changes during the course of the first pregnancy.

**Figure 2:**
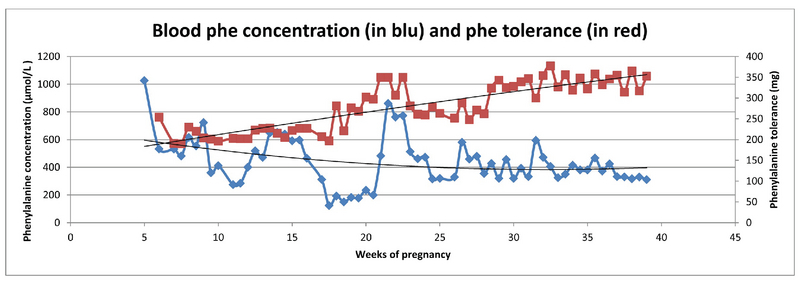
Phenylalanine tolerance changes during the course of the second pregnancy.

During the courses of the first and second pregnancies, the Phe tolerance increased 3.9- and 2.3-times, respectively (Table III). Table I and Figures 1 and 2 show what might not be considered as “satisfactory” metabolic control throughout pregnancies since a huge number of assessments of phenylalanine concentration was above the target range. Tables IV and V present a puzzling picture of the lack of association between maternal weight gain, as well as fetal weight gain, and the phenylalanine tolerance changes.

**Table 1:** Clinical data on singleton pregnancies of a PKU patient resulting in live births


	**PKU patient (p.R408W /p.R408W)**	
	**Pregnancy 1**	**Pregnancy 2**
Patient's age at conception [yrs]		28	32
In vitro fertilization [Y/N]		N	N
Smoking during pregnancy [Y/N]		N	N
Pre-pregnancy weight [kg]		88	90
Pre-pregnancy BMI [kg/m${}^{2}$]		33.8	34.6
Pregnancy weight gain [kg]		16	13
BMI before delivery [kg/m${}^{2}$]		40	39.6
Weight gain in the first trimester [kg]		6.0	1.0
BMI in the first trimester [kg/m${}^{2}$]		36.1	35.0
Newborn weight as a proportion of maternal weight gain [%]		20	30
Preconceptional Phe level [µmol/L]		1272 (21.2 mg%)	1495 (24.9 mg%)
Gestational age when diet initiated [weeks]		6	5
Dietary formula*		XP Maximum	PKU express 20
% of Phe assessments > 360 µmol/L (6mg%) during the whole pregnancy		40	58
% of Phe assessments < 120 µmol/L (2mg%) during the whole pregnancy		2	0
Daily protein intake from dietary formula [mg,g]*	14 Hbd	95.5	90
	28 Hbd	119.5	95
	34 Hbd	130	95
	37 Hbd	130	95
Daily energy intake [kcal]	14 Hbd	1868-2268	1712-2099
	28 Hbd	2483-2820	1850-2022
	34 Hbd	2300-2500	1915-2203
	37 Hbd	2500-2700	2102-2368
PKU: Phenylketonuria Y/N: Yes/No BMI: Body mass index			
Phe: Phenylalanine *to achieve optimal Phe control, natural sources of dietary proteins were greatly restricted			

**Table 2:** Birth measurements for completed pregnancies of the presented PKU patient


**Pregnancy no.**	**Weeks gestation**	**Gender [M/F]**	**Weight [g (percentile)]**	**Length [cm (percentile)]**	**Head circumference [cm (percentile)]**	**Apgar score**	**Congenital anomalies, dysmorphic features [Y/N]**	Phe concentration${}^{*}$[µmol/L]	**PKU-affection [Y/N]**
1	37	M	3030 (> 50)	54 (> 97)	34 (> 50)	10	N	109 (1.8 mg%)	N
2	39	M	4040 (> 90)	55 (> 97)	35 (> 50)	10	N	90 (1.5 mg%)	N
PKU: Phenylketonuria Phe: Phenylalanine M/F: Male/female									
*Whole blood phenylalanine concentration at newborn screening									

**Table 3:** The increase in phenylalanine tolerance${}^{1}$ during the course of pregnancies


**Pregnancy no.**	Phenylalanine tolerance increase${}^{*}$							
	**Trimester 1**		**Trimester 2**		**Trimester 3**		**During the whole pregnancy**	
	**mg**	**%**	**mg**	**%**	**mg**	**%**	**mg**	**%**
1	165 (120→285)	137	178 (204→382)	87	169 (298→467)	56	347 (120→467)	289
2	69 (178→247)	39	167 (181→348)	92	168 (237→-405)	70	227 (178→405)	128
${}^{*}$Based on average diet reconstructions								

**Table 4:** Estimated phenylalanine tolerance and its ratio to the weight of the pregnant woman in the first and second pregnancies


**Weeks gestation**	**Pregnancy 1**			**Pregnancy 2**		
	Phe level${}^{*}$ [µmol/L]	Phe tolerance${}^{**}$[mg]	Phe/weight${}^{***}$ [mg/kg]	Phe level${}^{*}$ [µmol/L]	Phe tolerance${}^{**}$ [mg]	Phe/weight${}^{***}$ [mg/kg]
7	964	157	1.69	504	190	2.03
14	424	293.5	3.12	646	204	2.25
28	302	318.5	3.19	391	305	3.11
37	261	439.5	4.23	331	314	3.02
39		368	350.5	3.40
Phe: Phenylalanine						
Data are presented as: *average of at least two assessments; **average of minimum two diet reconstructions; ***ratio of tolerated amount of Phe to the weight of the pregnant woman						

**Table 5:** Estimated phenylalanine tolerance and its ratio to fetal weight in the first and second pregnancies


Weeks gestation	**Pregnancy 1**			**Pregnancy 2**		
	Blood Phe level${}^{*}$ [µmol/L]	Daily Phe tolerance${}^{**}$ [mg]	Daily Phe tolerance/ fetal weight${}^{***}$ [mg/kg]	Blood Phe level*[µmol/L]	Daily Phe tolerance${}^{**}$ [mg]	Daily Phe tolerance/fetal weight${}^{***}$ [mg/kg]
22	278	309 (297-320)	617 (597-640)	767	320 (281-360)	641 (562-720)
25	337	310 (263-357)	413 (351-476)	320	263 (237-289)	351 (316-385)
29	360	338 (309-367	282 (257-306)	390	343 (308-379)	286 (257-316)
30	379	333 (312-354)	238 (223-253)	354	338 (323-354)	242 (231-253)
31	453	374 (327-422)	234 (204-264)	462	333 (300-366)	208 (187-229)
32	319	345 (324-367)	192 (180-204)	333	364 (300-402)	202 (167-223)
33	301	383 (350-417)	192 (175-208)	338	355 (306-405)	178 (153-202)
34	351	423 (397-450)	188 (176-200)	398	334 (301-367)	148 (134-163)
35	257	387 (343-423)	154 (137-169)	424	330 (275-386)	132 (110-154)
36	315	398 (359-438)	148 (133-162)	398	342 (318-367)	127 (118-136)
37	261	439 (412-468)	149 (140-159)	332	354 (310-399)	120 (105-135)
38		324	336 (293-380)	107 (93-121)
39		368	350 (316-385)	105 (94-115)
Phe: Phenylalanine						
Data are presented as: ${}^{*}$average of minimum two assessments; ${}^{**}$average of at least two diet reconstructions; ${}^{***}$ratio of the tolerated amount of Phe to the estimated weight of fetus						

### Ethical consideration

The patient had given a written consent before participating in the program of care for pregnant PKU patients developed by the Polish PKU Consensus.

## 3. Discussion

Poor reproductive outcome is observed in PKU women when dietary treatment is started late or when blood Phe levels remain elevated. Hyperphenylalaninemia in women may affect amino acid composition of uterine fluid (7). Maillot and colleagues (5) demonstrated that the proportion of time when the Phe concentrations in blood are within the target range is greater in pregnant PKU patients who started diet preconceptionally than postconceptionally. Ideally, all PKU patients wishing to become pregnant should do so in a planned way. Unfortunately, both pregnancies in our patient were unplanned. In Poland, in the general population, a high number of conceptions is unplanned (8, 9).

It has been demonstrated that after unsatisfied pregnancy dietary treatment, infants have commonly smaller anthropometric measurements at birth as well as impaired subsequent cognitive development (2, 3, 5). In our case, the high number of Phe assessments above 360 µmol/L was mostly not caused by a lack of patient's compliance and we observed normal neurodevelopment of patient's offspring. The Phe blood levels exceeded the desirable upper limit in spite of a low Phe intake similar to the case of PKU mother with PKU-affected fetus described by Kohlschütter and colleagues (10).

We suspect that the patient's low Phe tolerance might be related to her sever PKU phenotype (11) and the high prepregnancy BMI, as well as the high BMI during the course of pregnancies. Early body fat percentage strongly influence maternal and fetal health (12). In the study by MacLeod and colleagues (13), efforts to increase and maintain lean weight seem to increase the Phe tolerance in adult (non-pregnant) PKU patients. However, to our knowledge, there have been no studies indicating an effect of high preconceptional BMI and high BMI during the course of pregnancy on the limitation of Phe tolerance increase.

In the present case, good metabolic control was not achieved even in late pregnancy, time when increased phenylalanine requirement is expected due to the fetal growth and its phenylalanine hydroxylase activity in developing liver tissue (3, 6, 10, 14, 15). There was no impacting increase of Phe tolerance expressed as mg/kg of pregnant woman's body weight (b.w.) in both the pregnancies of our patient. In 1988, Hyánek and colleagues (15) reported mean increase of Phe tolerance 3 mg/kg b.w. (26%) during the last three months of pregnancy in a group of five women with PKU (six pregnancies), who returned to a low phenylalanine diet from the 5th or 6th month of pregnancy. The Phe tolerances in the first, second, and third trimesters of three PKU women (diet before conception, six and eight wk gestation) from the New England Maternal PKU Project were 450 mg (10 mg/kg b.w.), 720 mg (14 mg/kg b.w.), 1,300 mg (24 mg/kg b.w.); 800 mg (9mg/kg b.w.), 1,000 mg (14 mg/kg b.w.) 1,400 mg (14 mg/kg b.w.), and 675 mg (11 mg/kg b.w.), 1,300 mg (19 mg/kg b.w.), 1,500 mg (19 mg/kg b.w.), respectively (16). In one case, where the dietary treatment started 10 wk before conception, Thompson and colleagues (17) showed an increase of Phe tolerance from 6 mg/kg b.w. up to 30 mg/kg b.w. In our previous study, in two singleton pregnancies of PKU women (PKU genotypes: p.Q383X/p.R408W and p.R281L/p.R408W) on diet initiated preconceptionally, the mean Phe tolerance increased 12.1 mg/kg (172%) from 14 gestation wk to the pre-delivery week (18). Interestingly, the American College of Medical Genetics Guidelines on PKU (ACMG Practice Guidelines) recommended relatively high Phe intake for pregnant phenylketonurics: 265-770 mg in trimester 1, 400-1650 mg in trimester 2, and 700-2275 in trimester 3 (2), although based on the study of 23 pregnant patients with good metabolic control, Acosta *et al*. (19) provided data on the mean Phe tolerance in trimesters 1, 2, and 3 of 609 ± 220mg, 824 ± 411mg, and 1248 ± 513mg, respectively. The PKU patient (p.R408W/p.R261Q) reported by Duran and colleagues (20), who began the Phe-restricted diet at 13 wk of gestation had Phe tolerance of 300 mg in the second trimester. In two PKU patients (PKU genotypes: p.194del/p.P281L and p.165T/p.R408W) described by Kohlschütter and colleagues (10) in trimester 3, the Phe tolerance increased 900 mg (100%) and 500 mg (50%), respectively. Interestingly, in a pregnancy of the patient with the genotype p.R408W/p.R261Q with a fetus homozygous for PKU (genotype: p.R408W/p.R408W), the Phe tolerance increase was approximately 200 mg in trimester 3, and during the course of whole pregnancy, the Phe intake could not be increased above 400-500 mg/day without risking high Phe blood concentrations (10), which was majorly below the lowest recommended value of Phe intake for trimester 3 as per the ACMG Practice Guidelines (2). In the European guidelines on PKU (3), there is a suggestion based on the Kohlschütter and colleagues study (10) that” a low Phe tolerance in the third trimester of (treated) pregnancy may indicate foetal PKU.” Levy and colleagues (21) compared sibs with or without PKU from untreated pregnancies in phenylketonuric mother and observed no effect of the fetal genetic PKU status on maternal metabolic control as well as offspring outcomes. We would like to stress that described PKU woman gave birth to two healthy children and the Phe intake could not be increased above 470 mg/day in both the pregnancies; the increase in the Phe tolerance in the third trimester was below 200 mg. In the patient presented by us, the late third trimester Phe tolerance in the second pregnancy was 15% lower compared to the first pregnancy. It is known that the Phe tolerance may vary highly even between pregnancies in the same patient (3). Hyánek and colleagues (15) reported the Phe tolerance in two pregnancies of a PKU patient before conception and during the 5th, 6th, 8th, and 9th month of pregnancy: 10.0, 11.0, 13.0, 15.0, 15.5 mg/kg b.w. and 6.8, 7.8, 9.0, 10.0, 11.0 mg/kg b.w., respectively.

## 4. Conclusion

In conclusion, both the pregnancies of the presented PKU patient were difficult to manage because the expected increase of Phe tolerance did not take place. This case report does not confirm the observation that a lack of impacting Phe tolerance increase in pregnancy is a marker of fetal PKU affection.

##  Conflict of Interest

The authors deny any conflict of interest related to this study.
